# Orthoptic Changes following Photorefractive Keratectomy

**Published:** 2011-04

**Authors:** Zhale Rajavi, Nader Nassiri, Monir Azizzadeh, Alireza Ramezani, Mehdi yaseri

**Affiliations:** Ophthalmic Research Center, Shahid Beheshti University of Medical Sciences, Tehran, Iran

**Keywords:** Orthoptic Changes, Photorefractive Keratectomy, Strabismus

## Abstract

**Purpose:**

To report orthoptic changes after photorefractive keratectomy (PRK).

**Methods:**

This interventional case series included 297 eyes of 150 patients scheduled for PRK. Complete ophthalmologic evaluations focusing on orthoptic examinations were performed before and 3 months after PRK.

**Results:**

Before PRK, 2 (1.3%) patients had esotropia which remained unchanged; 3 (2%) patients had far exotropia which improved after the procedure. Of 12 cases (8%) with initial exotropia at near, 3 (2%) cases became orthophoric, however 6 patients (4%) developed new near exotropia. A significant reduction in convergence and divergence amplitudes (P < 0.001) and a significant increase in near point of convergence (NPC) (P < 0.006) were noticed after PRK. A reduction ≥ 10 PD in convergence amplitude and ≥ 5 PD in divergence amplitude occurred in 10 and 5 patients, respectively. Four patients had initial NPC > 10 cm which remained unchanged after surgery. Out of 9 (6%) patients with baseline stereopsis > 60 seconds of arc, 2 (1.33%) showed an improvement in stereopsis following PRK. No patient developed diplopia postoperatively.

**Conclusion:**

Preexisting strabismus may improve or remain unchanged after PRK, and new deviations can develop following the procedure. A decrease in fusional amplitudes, an increase in NPC, and an improvement in stereopsis may also occur after PRK. Preoperative evaluation of orthoptic status for detection of baseline abnormalities and identification of susceptible patients seem advisable.

## INTRODUCTION

Refractive errors are one of the most common causes of reduced vision in different populations.^1^–^4^ The National Health Interview Survey reported that 51.7% of people above 3 years of age wear glasses or contact lenses for optical reasons.^5^ Keratorefractive surgery (KRS) has been considered as a suitable alternative optical therapy and approximately 1.5 million KRS procedures are performed throughout the world each year.^6^ However, these procedures entail certain complications such as binocular diplopia, which may occur in patients with no history of strabismus and persist for a long time.^6^–^9^

Diplopia following KRS may stem from preoperative conditions such as anisometropia, heterotropia, lack of fusion and monofixation, or be due to problems after the operation such as under-, over-, or aniso-correction. However, no definite cause may be identified for this complication.^6^,^7^ Most of the available information in this field are based on case reports describing orthoptic problems after KRS in patients who had not undergone a complete evaluation preoperatively. To the best of our knowledge, only few studies with a limited number of cases have been published regarding the orthoptic condition of patients before and after KRS.^8^ Performing pre- and postoperative orthoptic examinations on a relatively large sample, we conducted this study to evaluate orthoptic changes and complications following photorefractive keratectomy (PRK), and to identify potential risk factors.

## METHODS

This interventional case series included 297 eyes of 150 patients undergoing PRK from July 2008 to October 2009. Exclusion criteria included age less than 18 years, corneal opacity, keratoconus or keratoconus suspect, moderate to severe dry eye, diabetes mellitus and collagen vascular diseases. Patients who provided informed consent were enrolled consecutively. This study was approved by the Review Board/Ethics Committee of the Ophthalmic Research Center at Shahid Beheshti University of Medical Sciences.

Before and 3 months after PRK, all subjects underwent a complete ophthalmologic evaluation focusing on orthoptic examinations by the same examiner using the same technique. Uncorrected and best corrected visual acuity (UCVA and BCVA) were measured using a Snellen chart at 6 meters. Visual acuity levels were converted to logarithm of minimum angle of resolution (logMAR) notations for statistical analyses.

Manifest refraction was performed objectively using an autorefractometer (Nidek, ARK 700, Gamagori, Japan) and refined subjectively considering maximum plus or minimum minus lenses. Cycloplegic refraction was determined 45 minutes after instillation of 1 drop of cyclopentolate (1%) and tropicamide (1%) 5 minutes apart using the same device. In hyperopic eyes, absolute hyperopia (minimum plus lens to achieve 20/20 vision), facultative hyperopia (maximum plus lens to maintain 20/20 vision) and latent hyperopia [cycloplegic refraction – (absolute hyperopia + facultative hyperopia)] were calculated. Amblyopia was defined as a 2-line difference in BCVA in fellow eyes, or BCVA less than 20/30 in either eye. Eyes were classified as simple myopic, myopic astigmatic, simple hyperopic, hyperopic astigmatic, and astigmatic based on refractive errors being equal or greater than 1 diopter (D) in spherical or astigmatic power.

All patients were examined for ocular deviations using the alternate prism cover test with optimal spectacle correction at both near and distance. The patients were considered strabismic if esodeviation was ≥ 4 prism diopters (PD) or exodeviation was ≥ 8 PD. Presence of A or V pattern (≥ 15 PD difference between deviation in primary position, and up or down gaze) and high AC/A (> 3) were assessed preoperatively. Extraocular motility was evaluated in all gaze positions to determine any over- or underaction of extraocular muscles. Convergence and divergence amplitudes were measured by bar prisms and break points were recorded in PD. Loss of convergence and divergence amplitude exceeding 10 and 5 PD respectively was considered as significant. Fusional amplitude (convergence + divergence amplitudes) ≤ 10 PD was considered as a risk factor for deviation. Near point of convergence (NPC) ≥ 10 cm and stereoacuity (evaluated by Titmus test) > 40 seconds of arc were considered as abnormal.

Anterior and posterior segment evaluations were performed by slitlamp biomicroscopy and indirect ophthalmoscopy. Intraocular pressure was measured by Goldmann applanation tonometry. Preoperatively, ancillary tests including computed topography, Orbscan II (Bausch & Lomb, Rochester, NY, USA) and pachymetry (Tomy SP 2000, Cambridge, MA, USA) were performed.

All patients underwent PRK with the Technolas Z-217 excimer laser machine (Bausch & Lomb, Rochester, NY, USA) under topical anaesthesia using tetracaine eye drops (0.5%). Fixation was paralleled with target lights and an 8-mm circle of corneal epithelium was removed from the center mechanically. Rechecking patient fixation, laser ablation was performed based on the amount of objective and subjective refractions. In cases with myopia higher than −3.75 D, the ablated area was kept in contact with a mitomycin C sponge (0.2 mg) for 20 seconds and then irrigated with 50 mL normal saline solution. Bandage lenses were fitted for all cases.

Postoperatively, all patients received chloramphenicol 0.1% and diclofenac sodium 0.1% eye drops four times a day for 10 and 5 days, respectively. Betamethasone 0.1% drops were prescribed four times a day for 5 days which was then replaced by fluorometholone 0.1% eye drop for the following 2 months. The bandage contact lenses were kept in place for 3 to 5 days depending on epithelial healing.

Refractive results were expressed as over- and undercorrection (≥ 1 D), astigmatism axis rotation (> 20 degrees of astigmatic power ≥ 1 D), new astigmatism formation (≥ 1 D), and anisometropia (≥ 1 D). Statistical analyses were performed using SPSS 15.0 software. Frequency values were compared using Chi-square (or Fisher exact) test and mean values were compared using paired t-test. P-values < 0.05 were considered as statistically significant.

## RESULTS

One hundred and fifty patients including 46 (30.7%) male and 104 (69.3%) female subjects with mean age of 27.6 ± 7.6 (range, 19 to 59) years were enrolled. All patients were operated bilaterally, except 3 due to emmetropia on one side. Upon presentation, 173 (58.2%) eyes had simple myopia, 76 (25.6%) had myopic astigmatism, 19 (6.4%) had simple hyperopia, 10 (3.4%) had hyperopic astigmatism, and 19 (6.4%) had astigmatism. Three eyes were amblyopic.

Mean postoperative UCVA (0.02 ± 0.07 logMAR) was not significantly different from mean preoperative BCVA (0.01 ± 0.05 logMAR) (P = 0.324). The difference between pre- and postoperative mean spherical equivalent in each subtype of the abovementioned refractive errors was statistically significant (P < 0.001 for all comparisons). Postoperatively, 247 (83.2%) eyes became emmetropic; however, over- and undercorrections were encountered in 42 (14.1%) and 8 (2.7%) eyes, respectively. Preoperatively 23 patients (15.3%) were anisometropic; after PRK, only 2 (1.3%) cases of anisometropia were detected. Six eyes (4%) of 4 patients developed new astigmatism after PRK the amount of which was 1.0 D in 5 eyes and 1.25 D in 1 eye. Rotation of astigmatic axis occurred in 7 eyes of 5 patients.

### Deviation

Before surgery, one patient (0.66%) had 12 PD esotropia both at far and near, and another patient had 4 PD esotropia only at near. Deviations in these patients remained unchanged postoperatively; both patients were hyperopic and became emmetropic after surgery. New esodeviation did not develop in any of the patients after PRK.

Two patients (1.3%) had exotropia ≥ 8 PD at far and near (#49 and 106) and one (#35) only at far before PRK (Table 1). Deviations in these patients improved postoperatively. All of these patients were myopic; of these 2 became emmetropic and one (#35) became overcorrected. No case of new far exotropia developed after the procedure.

Preoperatively, exotropia ≥ 8 PD was detected at near in 12 patients (8%). Three cases (#19, 21 and 64) became orthophoric (< 8 PD) and 9 patients remained unchanged following surgery. Six other patients (4%) (#1, 17, 25, 28, 42 and 120) developed new exotropia ≥ 8 PD at near after PRK (Table 2). All of these subjects became emmetropic; however 3 of them (#1, 17 and 120) lost more than 10 PD of convergence amplitude after PRK.

A or V pattern, high AC/A, or over- and under-action of extraocular muscles were not detected in any of the patients pre- or postoperatively. None of the patients in this study complained of diplopia after PRK.

### Fusion

Mean convergence amplitude was reduced from 20.7 ± 7.0 PD at baseline to 18.3 ± 5.9 PD after PRK (P < 0.001). Convergence amplitude in 10 patients (6.66%) decreased ≥ 10 PD after the operation; 3 of them (#1, 17 and 120) developed new near exodeviation (Table 3). Mean divergence amplitude decreased from 9.5 ± 4.1 PD at baseline to 8.6 ± 3.5 PD after PRK (P < 0.001). Divergence amplitude in 5 patients (3.33%) was reduced ≥ 5 PD; nonetheless none of them developed new esodeviation (Table 4).

Mean fusional amplitude decreased from 30.14 ± 9.0 at baseline to 26.84 ± 7.6 PD after PRK. One patient (#96) had fusional amplitude of 8 PD before PRK which remained unchanged thereafter (Table 1). Fusional amplitude in two other cases (#17 and 120) demonstrated significant reduction from 22 PD at baseline to 10 PD postoperatively. This reduction occurred primarily in convergence amplitude and was associated with new near exodeviation (Table 2). Both patients were hyperopic preoperatively and became emmetropic after PRK.

Mean NPC increased from 5.22 ± 1.45 cm at baseline to 5.39 ± 1.4 cm after the procedure (P = 0.006). There were 4 patients (#17, 26, 28 and 120) with NPC > 10 cm before and after PRK. Of these subjects 2 were simple myopic and 2 others were simple hyperopic; all of them became emmetropic after surgery except one patient (#26), who developed new astigmatism. The other 3 subjects developed new near exodeviation postoperatively. None of the patients with normal preoperative NPC developed abnormal NPC after PRK.

### Stereopsis

There were 9 (6%) patients with stereopsis worse than 60 seconds of arc before surgery. Of these, stereopsis improved from 200 to 40 seconds of arc after PRK in 2 (1.33%) cases (#54 and 100, Table 5). Both had hyperopic astigmatism preoperatively and became emmetropic after PRK. In the remaining 7 patients, stereopsis remained unchanged. Of these, 3 subjects (#96, 104 and 137) had eye deviations, 2 (#107 and 124) had high astigmatism preoperatively, and 2 others (#17 and 120) developed new near exodeviation with > 10 PD loss of convergence amplitude. Emmetropia was achieved in all of these patients.

## DISCUSSION

This interventional case series demonstrated that PRK neither improved nor induced esodeviations. Regarding exodeviation however, 6 (4%) patients showed improvement and 6 (4%) developed new deviation. A noticeable decrease in convergence and divergence amplitudes was detected in 10 (6.6%) and 5 (3.3%) patients, respectively; but only reduction in convergence amplitude resulted in new exodeviation. Stereopsis did not change significantly after PRK, although 2 (1.3%) patients experienced an improvement in binocular vision.

The success rate of PRK was acceptable in our study, such that emmetropia (±1.00 D) was achieved in 83.2% of cases which is comparable to similar studies.^10^,^11^ Furthermore, 82.2% of eyes reached final UCVA of ≥ 20/20 which is similar to corresponding figures in other reports.^11^–^13^

None of our patients, even those with new postoperative deviations developed diplopia after PRK. Godts et al^7^ reported 5 patients with diplopia after KRS, of whom 4 had strabismic problems before surgery including latent cranial nerve VI palsy, intermittent deviation, and previous strabismus surgery. The authors recommended more caution about strabismic problems among individuals seeking KRS. None of our patients had extraocular muscle paresis or previous strabismus surgery prior to the operation.

Kushner et al^6^ reported two patients with diplopia after KRS who had astigmatism axis rotation > 30 degrees. The authors believed that the diplopia was due to image tilt and an optically induced cyclotropia as documented by Maddox rod test. These patients had no deviation or objective fundus torsion but could not fuse due to the induced astigmatism. In our study, 7 (2.35%) eyes had axis rotation ≥ 20 degrees after PRK; however, none of them complained of diplopia, mainly because of the low power of residual astigmatism (maximum, 1.25 D) and lack of anisometropia. Evaluating the reasons for post-KRS diplopia in 28 patients, Kushner et al^6^ suggested five mechanisms including: technical problems, prior deviations, aniseikonia, iatrogenic monovision and improper control of accommodation. Other proposed mechanisms for post-KRS diplopia include monocular versus binocular fixation, postural changes, and subjective rotation.^8^

In our study, 2 patients (1.33%) had esotropia preoperatively, including one case of partially accommodative esotropia of 12 PD with full correction (+5.00 OU), and a patient with orthophoria at far with full correction (+4.00 OU) but 4 PD esotropia at near. Although both subjects became emmetropic after PRK, no change occurred in the amount of deviations. Sabetti et al^14^ reported a reduction in accommodative esotropia in all of their 18 patients except one who had recurrence of hyperopia and deviation 2 years after KRS. Kowal et al^8^ believed that the success of KRS in hyperopic patients with accommodative esotropia depends on the amplitude of accommodation, residual hyperopia and ability of fusion.

In our study, 6 out of 15 patients (40%) with initial exotropia improved postoperatively. All of them were myopic which was completely corrected after PRK except in one case, who developed an overcorrection. None of these subjects developed reduced convergence amplitudes after the operation. The improvement in deviation in previously myopic patients might be due to the additional need for accommodation and convergence after becoming emmetropic especially in patients who have not used glasses for near vision before surgery. Godts et al^9^ reported a shift to intermittent exotropia in 2 of 6 exotropic subjects resulting in improvement in binocular vision. Nemet et al^15^ also reported two anisomyopic cases with exotropia whose deviations were corrected after KRS.

In our study, 6 patients (4%) developed new near exotropia following PRK. Of these, 4 had myopia, near exophoria < 8 PD and a rather low convergence amplitude preoperatively. Their convergence amplitude was further reduced postoperatively, ranging from 2 to 13 PD causing conversion of latent exophoria to manifest exotropia. The other 2 individuals with new near exotropia after PRK were initially hyperopic which was entirely corrected. Hyperopic correction may be accompanied with further relaxation of accommodation causing exotropia in a patient with weak binocular fusion. Godts et al^9^ emphasized on informing patients presenting with latent strabismus about the possibility of developing manifest deviations after KRS due to a decrease in fusional amplitudes.

We observed a significant reduction in convergence amplitude after PRK in our patients. Although the mean amount of this reduction (about 2.5 PD) was clinically insignificant, 10 cases lost more than 10 PD of convergence amplitude which may be significant. Three of these patients, even developed new near exotropia. Although none of these patients complained of near diplopia, the development of new deviations emphasizes the possibility of visual symptoms even after successful KRS, especially in patients with low convergence amplitude at presentation. Kushner et al^6^ considered fusional amplitudes > 10 and < 5 PD as good and poor fusional reserves, respectively. None of our patients had initial fusion amplitudes less than 16 PD.

In our patients, mean divergence amplitude decreased significantly after PRK. Although the mean amount of reduction (about 1 PD) does not seem significant, a noticeable decrease (> 5 PD) in divergence amplitude occurred in 5 subjects. Even in these cases, however, no case of new esotropia was observed. Considering the manifestation of exodeviation in 3 out of 10 patients with loss of convergence amplitude, one may conclude that a decrease in convergence amplitude is more significant than that of divergence amplitude in terms of predisposing to ocular misalignment after KRS. This conclusion should be made with caution because of the small number of cases in our series developing such complications.

The increase in NPC (about 0.17 cm) after PRK in our series was statistically but not clinically significant. The reason may be the decrease in mean convergence amplitude in our series. Four cases had abnormal NPC (> 8 cm) postoperatively but all had weak convergence before PRK. Additionally, they had near exophoria preoperatively which may further deteriorate this condition.

Evaluating the results of KRS on 13 adult strabismic patients, Godts et al^9^ noticed no change in stereopsis except in 2 cases in whom binocular function and consequently stereopsis improved due to conversion of manifest deviation to latent phoria after surgery. In children with amblyogenic refractive errors, binocular function after KRS was achieved in 7% to 78% of subjects as reported by Tychsen et al.^10^ Although mean stereopsis in our patients did not change significantly after PRK, 2 out of 9 cases with initial stereopsis worse than 80 seconds of arc showed a noticeable improvement (from 200 to 40 seconds of arc). Both of these patients had hyperopic astigmatism preoperatively and became emmetropic after the procedure. Of the 7 remaining patients without any change in stereopsis; 3 had eye deviation preoperatively which remained unchanged, 2 developed new near exotropia postoperatively, and 2 cases with high astigmatism (about 4 D) before surgery became emmetropic after PRK. Of these 7 cases, 5 were either hyperopic or hyperopic astigmatic. Based on the results of this study, most patients (78%) seeking KRS with stereopsis abnormalities are hyperopic, and stereopsis does not usually improve after surgical correction of the refractive error. However, an improvement in stereoscopic vision may occasionally be observed.

Most published reports in the field of post-KRS orthoptic problems are retrospective case series. To the best of our knowledge, the current study is the first prospective study focusing on orthoptic status in patients undergoing KRS before and after the procedure. However, limited sample size may be considered as a shortcoming in the current study. To demonstrate the exact incidence of each of the above mentioned orthoptic changes after KRS, prospective studies with large sample size are required.

In summary, despite the development of some new exodeviations and reduction in convergence and divergence amplitudes after PRK in a small number of our patients, none of them developed diplopia. Although the incidence of these complications was low in our study, considering the large number of people undergoing KRS around the world, the overall rate of such complications may be significant. We therefore recommend a careful orthoptic evaluation in addition to other ophthalmologic examinations before KRS to determine patients at risk of strabismic or fusional problems.

## Figures and Tables

**Table 1:** Pre- and postoperative orthoptic characteristics in patients with eye deviations before photorefractive keratectomy

No	Code	Sex	Age (yr)	Pre-operative								Post-operative							

				Refraction (D)		Deviation (PD)		CA (PD)	DA (PD)	NPC (cm)	ST (SOA)	Refraction (D)		Deviation (PD)		CA (PD)	DA (PD)	NPC (cm)	ST (SOA)
	
				OD	OS							OD	OS						
1	96	F	23	+5-0.5×155	+5-0.5×37	ET=12	ET’=12	4	4	5	200	+0.25-0.5×160	Plano-0.5×170	ET=10	ET’=10	4	4	5	200
2	104	F	50	+4-0.5×130	+4-0.25×90		ET’=4	25	8	5	100	+0.5-0.25×150	+0.25×90		ET’=4	25	8	5	100
3	19	M	24	−3.5-0.5×70	−3.5-0.5×144		XT’=16	16	8	8	40	+0.75-0.25×20	+0.75-0.5×30		XT’=5	16	8	6	40
4	21	F	26	−3.25-0.25×14	−3.5-0.75×8		XT’=8	25	6	3	40	Plano-0.25×10	Plano-0.25×5		XT’=4	25	6	4	40
5	32	F	24	−2.5-0.25×49	−2.5-1×134		XT’=12	18	14	5	40	Plano-0.5×60	Plano-0.25×90		XT’=14	10	12	5	40
6	35	F	24	−2.75-0.5×135	−3.25-0.25×159	XT=8	XT’=2	12	14	3	40	+1-0.5×17	+1.5-0.25×150	XT=3		20	12	3	40
7	49	F	28	−5.25-0.5×160	−5.5-0.75×15	XT=8	XT’=14	12	12	7	40	+0.5-0.25×170	+0.5-0.25×15	XT=4	XT’=10	12	12	7	40
8	51	F	27	−2.75	−3.75		XT’=12	25	12	4	40	+0.25	+0.25		XT’=12	20	10	5	40
9	57	F	19	−5.25-0.5×95	−4.25-0.5×65		XT’=10	25	12	4	40	+0.5	+0.5		XT’=10	20	10	5	40
10	64	F	21	−7.5-1×140	−7-0.5×47		XT’=8	16	10	5	40	Plano-0.5×170	Plano	XT=4	XT’=6	16	10	6	40
11	69	M	23	−1.5-2×155	−3-0.75×10		XT’=10	12	12	6	40	+1-0.75×10	+0.25-0.5×180		XT’=8	12	8	6	40
12	79	F	21	−5.5-1×180	−4.75		XT’=10	18	8	6	40	−0.25-0.25×180	−0.75		XT’=10	16	8	6	40
13	80	M	37	−1.25	−0.5-1×170		XT’=14	14	6	6	40	+0.5	+0.25-0.25×180		XT=14	14	6	6	40
14	106	F	29	−2-0.5×180	−2-0.5×30	XT=8	XT’=8	8	8	6	40	+0.75-0.25×160	+0.5-0.25×30		XT’=8	8	8	6	40
15	117	F	27	−2.75	−3.75		XT’=12	25	12	4	40	+0.25	+0.25		XT’=12	20	10	5	40
16	137	F	36	+3	+3.5	XT=4	XT’=10	14	8	6	80	+0.25	−0.25	XT=4	XT’=10	12	8	6	80
17	141	M	23	−1.5-2×150	−3-0.75×175		XT’=10	12	12	6	40	+1-0.75×180	+0.25-0.5×180		XT’=8	12	8	6	40

M, male; F, female; OD, right eye; OS, left eye; yr, year; CA, convergence amplitude; DA, divergence amplitude; NPC, near point of convergence; ST, stereopsis; D, diopter; PD, prism diopter; SOA, second of arc; ET, esotropia at far; ET’, esotropia at near; XT, exotropia at far; XT’, exotropia at near.

**Table 2:** Pre- and postoperative orthoptic characteristics in patients with new eye deviations after photorefractive keratectomy

No	Code	Sex	Age (yr)	Refraction (D)							Post-operative							

				Pre-operative		Deviation (PD)	CA (PD)	DA (PD)	NPC (cm)	ST (SOA)	Refraction (D)		Deviation (PD)		CA (PD)	DA (PD)	NPC (cm)	ST (SOA)
	
				OD	OS						OD	OS						
1	1	M	39	−2.5-1×180	−2.5-2×150	XT’=3	25	6	5	40	+0.75-0.25×180	+0.75-0.25×180		XT’=10	12	6	7	40
2	17	F	48	+1.5-0.5×155	+1.5-0.5×134	---	16	6	10	80	−0.25-0.5×120	−0.25-0.5×85		XT’=8	6	4	10	80
3	25	F	29	−3.5-0.5×150	−3.5-0.5×160	XT’=5	18	4	5	40	+0.75-0.25×150	+0.75-0.5×160		XT’=10	16	4	6	40
4	28	F	22	−3.5-0.5×95	−3.25-0.5×40	XT’=4	20	6	10	40	+0.5-0.5×180	+0.25-0.5×80	XT=2	XT’=10	12	6	10	40
5	42	M	32	−0.25	−0.75-1.75×85	XT’=6	10	6	7	40	Plano-0.5×75	−0.5-0.75×100	XT=3	XT’=16	8	6	7	40
6	120	F	25	+1.5-0.5×155	+1.5-0.5×134	---	16	6	10	80	−0.25-0.5×125	−0.25-0.5×80		XT’=8	6	4	10	80

M, male; F, female; OD, right eye; OS, left eye; yr, year; CA, convergence amplitude; DA, divergence amplitude; NPC, near point of convergence; ST, stereopsis; D, diopter; PD, prism diopter; SOA, second of arc; XT, exotropia at far; XT’, exotropia at near.

**Table 3:** Pre- and postoperative orthoptic characteristics in patients with reduced convergence amplitude ≥ 10 PD after photorefractive keratectomy

No	Code	Sex	Age (yr)	Pre-operative								Post-operative							

				Refraction (D)		Deviation (PD)		CA (PD)	DA (PD)	NPC (cm)	ST (SOA)	Refraction (D)		Deviation (PD)		CA (PD)	DA (PD)	NPC (cm)	ST (SOA)
	
				OD	OS							OD	OS						
1	1	M	39	−2.5-1×180	−2.5-2×150		XT’=3	25	6	5	40	+0.75-0.25×180	+0.75-0.25×180		XT’=10	12	6	7	40
2	2	F	21	−2.75-0.75×100	−3.25-0.5×70	---		25	12	5	40	+1.25-0.5×90	+0.5-0.75×50	XT=3	XT’=2	12	12	5	40
3	4	M	26	−3.75-1.25×5	−4-1.5×179	XT=3		35	8	8	40	Plano-0.5×174	+1.25-0.5×156		XT’=3	25	8	8	40
4	6	F	51	+1.75-0.25×75	+2-1×151	---		40	20	5	40	−0.25-0.25×25	−0.25-0.5×150	---		20	10	5	40
5	7	F	30	−4.25-1×170	−4.25-1.5×170	---		35	18	3	40	+1.5-0.5×50	+1-0.25×175	---		25	8	3	40
6	17	F	48	+1.5-0.5×155	+1.5-0.5×134	---		16	6	10	80	−0.25-0.5×120	−0.25-0.5×85		XT’=8	6	4	10	80
7	27	F	28	−4.75-2×177	−4-0.5×8		XT’=6	35	12	3	40	+2-0.5×85	+2-0.75×55		XT’=5	20	10	3	40
8	116	F	28	−4.75-2×170	−4-0.5×10		XT’=6	35	12	3	40	+2-0.5×80	+2-0.75×60		XT’=5	20	10	3	40
9	120	F	25	+1.5-0.5×155	+1.5-0.5×134	---		16	6	10	80	−0.25-0.5×125	−0.25-0.5×80		XT’=8	6	4	10	80
10	132	M	26	−3.75	−3.5	XT=3	XT’=3	35	8	8	40	Plano-0.5×174	+1.25-0.25×156		XT’=3	25	8	8	40

M, male; F, female; OD, yr, year; right eye; OS, left eye; CA, convergence amplitude; DA, divergence amplitude; NPC, near point of convergence; ST, stereopsis; D, diopter; PD, prism diopter; SOA, second of arc; XT, exotropia at far; XT’, exotropia at near.

**Table 4:** Pre- and postoperative orthoptic characteristics in patients with reduced divergence amplitude ≥ 5 PD after photorefractive keratectomy

No	Code	Sex	Age (yr)	Pre-operative								Post-operative							

				Refraction (D)		Deviation (PD)		CA (PD)	DA (PD)	NPC (cm)	ST (SOA)	Refraction (D)		Deviation (PD)		CA (PD)	DA (PD)	NPC (cm)	ST (SOA)
	
				OD	OS							OD	OS						
1	3	M	40	−3.5	−3.75	XT=3	XT’=3	20	25	5	40	+0.75-0.5×80	+0.25	XT=7	XT’=5	12	12	4	40
2	6	F	51	+1.75-0.25×75	+2-1×150	---		40	20	5	40	−0.25-0.25×25	−0.25-0.5×150	---		20	10	5	40
3	7	F	30	−4.25-1×17	−4.25-1.5×150	---		35	18	3	40	+1.5-0.5×25	+1-0.25×150	---		25	8	3	40
4	16	F	40	+3.75-0.5×100	+3.75-0.75×157		XT’=4	18	16	5	60	+1.75-0.5×160	+1.25-0.75×160		XT’=4	18	10	8	50
5	24	F	22	−2.5-0.5×158	−1.25-0.5×175	---		18	12	5	40	+2-0.25×30	+1-0.25×50	---		10	6	8	40

M, male; F, female; yr, year; OD, right eye; OS, left eye; CA, convergence amplitude; DA, divergence amplitude; NPC, near point of convergence; ST, stereopsis; D, diopter; PD, prism diopter; SOA, second of arc; XT, exotropia at far; XT’, exotropia at near.

**Table 5:** Pre- and postoperative orthoptic characteristics in patients with stereopsis ≥ 60 seconds of arc before photorefractive keratectomy

No	Code	Sex	Age (yr)	Pre-operative								Post-operative							

				Refraction (D)		Deviation (PD)		CA (PD)	DA (PD)	NPC (cm)	ST (SOA)	Refraction (D)		Deviation (PD)		CA (PD)	DA (PD)	NPC (cm)	ST (SOA)
	
				OD	OS							OD	OS						
1	17	F	48	+1.5-0.5×155	+1.5-0.5×134	---		16	6	10	80	−0.25-0.5×125	−0.25-0.5×80		XT’=8	6	4	10	80
2	54	M	37	+4.25-2.5×175	+5.25-4×170	---		25	6	5	200	+0.25-0.25×100	+0.25-0.5×125	---		20	6	5	40
3	96	F	23	+5-0.5×157	+5-0.5×37	ET=12	ET’=12	4	4	5	200	+0.25-0.5×160	Plano-0.5×170	ET=10	ET’=10	4	4	5	200
4	100	M	37	+4.25-2.5×180	+5.25-4×170	---		25	6	5	200	+0.25-0.25×180	+0.25-0.5×130	---		20	6	5	40
5	104	F	50	+4-0.5×130	+4-0.25×90		ET’=4	25	8	5	100	+0.5-0.25×150	+0.25-0.25×90		ET’=4	25	8	5	100
6	107	M	34	−3.25	−1-3.25×150	---		40	12	5	400	+0.5-0.25×180	+0.25-0.5×180	---		38	12	5	400
7	120	F	25	+1.5-0.5×155	+1.5-0.5×144	---		16	6	10	80	+0.2.5-0.5×125	−0.25-0.5×80		XT’=8	6	4	10	80
8	124	M	20	−1.25-4.75×7	−1.75-3×160	---		25	16	5	140	+0.25-0.5×180	Plano-0.5×180	---		20	16	5	140
9	137	F	36	+3	+3.5	XT=4	XT’=10	14	8	6	80	+0.25	−0.25	XT=4	XT’=10	12	8	6	80

M, male; F, female; yr, year; OD, right eye; OS, left eye; CA, convergence amplitude; DA, divergence amplitude; NPC, near point of convergence; ST, stereopsis; D, diopter; PD, prism diopter; SOA, second of arc; XT, exotropia at far; XT’, exotropia at near.
